# Supplementary material for: The gate injection-based field-effect synapse transistor with linear conductance update for online training
Source: Nat Commun. 2022 Oct 28;13:6431. doi: 10.1038/s41467-022-34178-9 (PMC9616899; doi:10.1038/s41467-022-34178-9)
Supplement: Supplementary file 1 — Supplementary Information [file 41467_2022_34178_MOESM1_ESM.pdf]

Supplementary Information

The Gate Injection-based Field-Effect Synapse Transistor with Linear Conductance Update for Online Training

*Seokho Seo[1, †], Beomjin Kim[1, †], Donghoon Kim[1, †], Seungwoo Park[1, †], Tae Ryong Kim[1], Junkyu Park[1], Hakcheon Jeong[1], See-On Park[1], Taehoon Park[1], Hyeok Shin[1], Myung-Su Kim[1], Yang-Kyu Choi[1] and Shinhyun Choi[1, *]*

[1]The School of Electrical Engineering, Korea Advanced Institute of Science and Technology (KAIST), Daejeon 34141, Republic of Korea

[†]These authors contributed equally to this work.

***Address correspondence to** Shinhyun Choi, The School of Electrical Engineering, Korea Advanced Institute of Science and Technology (KAIST), Daejeon 34141, Republic of Korea, Email: shinhyun@kaist.ac.kr

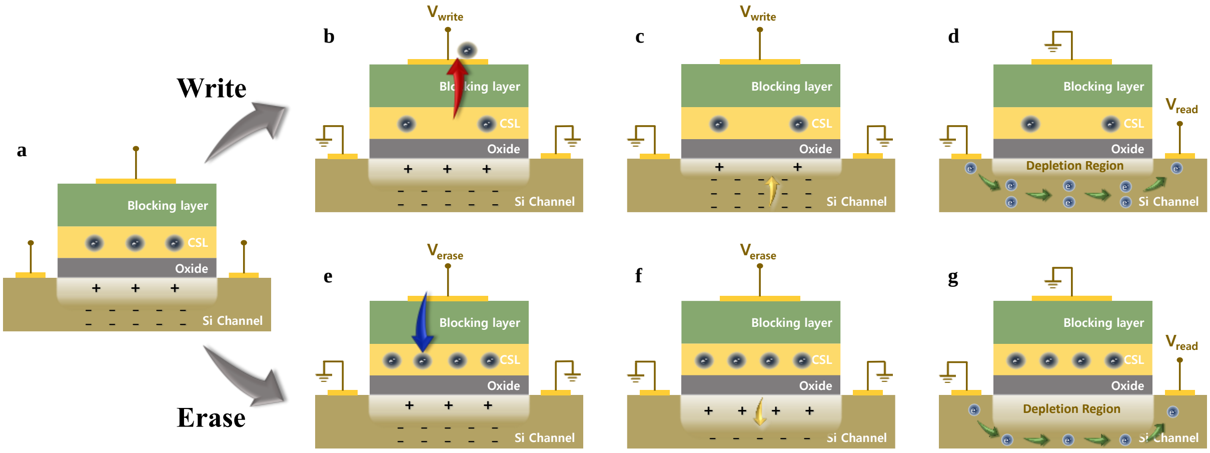

Supplementary Fig. 1 Detailed operation principle of the GIFET in 2D schematic diagrams. a Initial state. **b-d** Write operation sequence; **b** electron extraction, **c** reduction of depletion region, and **d** expansion of channel. **e-g** Erase operation sequence; **e** electron injection, **f** expansion of depletion region, and **g** reduction of depletion region.

GIFET does work in a depletion mode. The device was designed in the form of an n-n-n junctionless transistor, and detailed 2D schematic diagrams of the operation processes were prepared for a more accurate understanding, as shown in Supplementary Fig. 1.

For the erase process, a depletion layer was formed under the gate oxide in accordance with the electrons stored in the charge store layer (CSL) by applying a negative voltage to the gate stack. Because the electrons stored in the CSL are negative, the electrons in the n-type channel will be removed due to repulsive force, and the depletion region will be created in the channel.

For the write process, a positive bias was applied to the gate while the source and drain were grounded. Negatively charged electrons on the WO_x layer were extracted to the gate metal due to the electric field. Subsequently, it results in shrinking of the depletion region in the channel.

To read the stored weight of the device, the gate is grounded, and the read voltage is applied to the drain. The synaptic weight is obtained by measuring the resistance between the source and drain. Therefore, by controlling a portion of the depletion region in the channel, the synaptic weight can be stored.

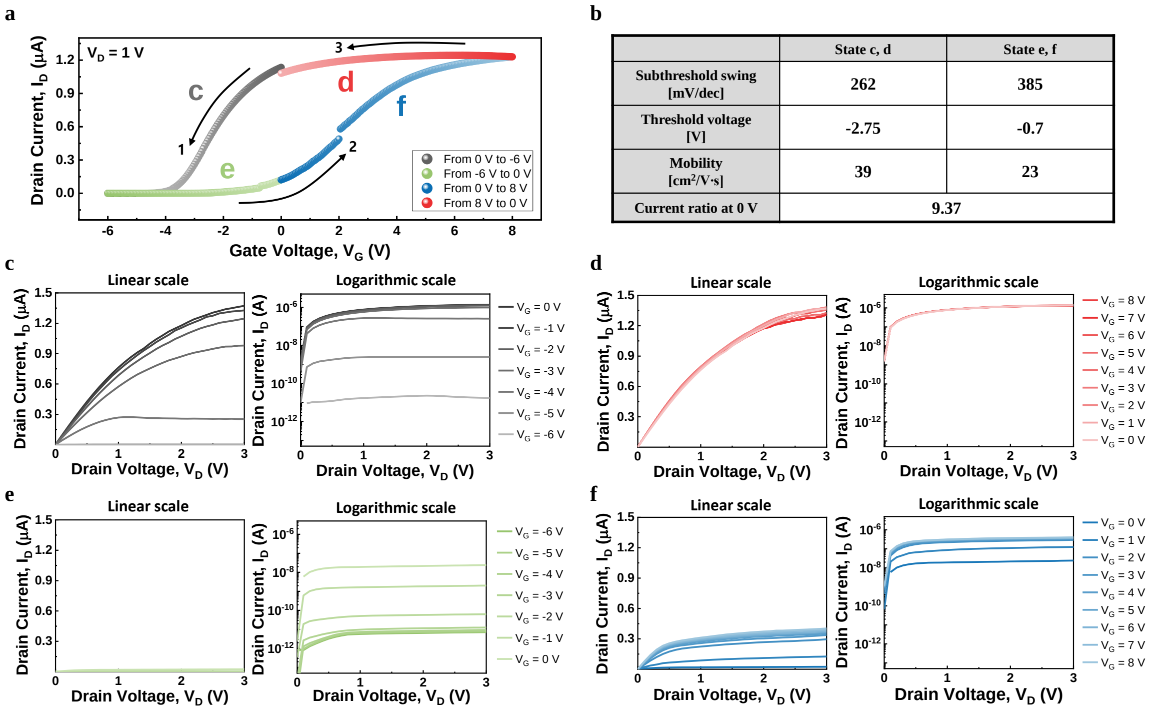

	State c, d	State e, f
Subthreshold swing [mV/dec]	262	385
Threshold voltage [V]	-2.75	-0.7
Mobility [cm²/V·s]	39	23
Current ratio at 0 V	9.37	

Supplementary Fig. 2 Transistor characteristics of GIFET. a I_D-V_G characteristic (V_D = 1 V). **b** Summary table of subthreshold swing, threshold voltage, mobility, and current ratio at 0 V of GIFET. **c-f** Output curves for each state in Supplementary Fig. 2a.

Subthreshold swing calculation

Subthreshold swing of GIFET was calculated using the following equation:

$$SS = \left(\frac{\partial(\log I_\mathrm{D})}{\partial V_\mathrm{G}}\right)^{-1}$$

We calculated each of the states and selected the minimum value.

Mobility calculation

Mobility was calculated using the following equation:

$$\mu = \frac{\mathrm{d}I_\mathrm{D}}{\mathrm{d}V_\mathrm{G}} \cdot \frac{L}{W C_\mathrm{t} V_\mathrm{D}}$$

dI_D/dV_G (transconductance, g_m) was measured through I_D-V_G characteristic measurements, L is the channel length (20 μm), W is the channel width (5 μm), C_t (5.86×10^{-4} F/m^2) is the total capacitance between the gate metal and the channel per unit area ($C_t=(d_{oxide}/(\varepsilon_0\times\varepsilon_{oxide}) + d_{CSL}/(\varepsilon_0\times\varepsilon_{CSL}) + d_{BL}/(\varepsilon_0\times\varepsilon_{BL}))^{-1}$, $\varepsilon_0=8.85\times10^{-12}$ F/m^2, ε_{oxide}, ε_{CSL}, and ε_{BL} are the dielectric constants of SiO_2 (3.9)[1], WO_x (5)[2], and a-Si (13.5)[3], respectively. d_{oxide}, d_{CSL}, and d_{BL} are the thicknesses of the oxide layer (25 nm), charge store layer (25 nm), and blocking layer (50 nm), respectively). V_D is 1 V. The mobility in Supplementary Fig. 2b was selected as the maximum value.

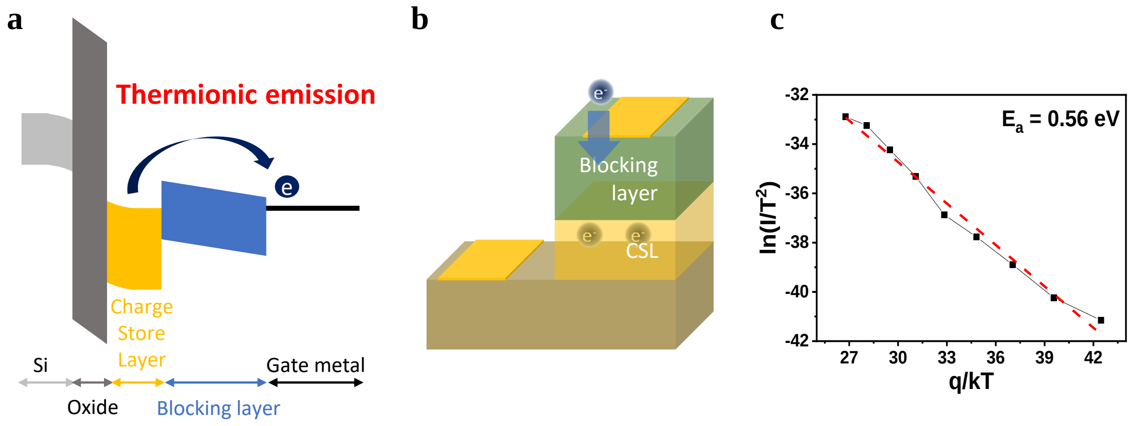

Supplementary Fig. 3 Comparison of barrier heights between write and erase operations. **a** Band diagram of GIFET under applying positive bias on the gate metal. Electrons are extracted from the charge store layer to the gate metal, and this operation is dominantly affected by the barrier between the charge store layer and the blocking layer. **b** Schematic of gate stack without gate oxide. The arrow indicates the direction of movement of electrons under applying negative bias on the gate metal, as in the case of erase operation. **c** Arrhenius plot for **b** under varied temperature from 273 K to 423 K when applied voltage is -1 V. Activation energy is dominantly derived between the gate metal and blocking layer.

GIFET is designed to have a difference in barrier height for the write and erase operations. During the write operation in Supplementary Fig. 3a, electrons are extracted from the CSL to the gate through the blocking layer. The barrier height formed by the CSL and blocking layer plays an important role in the conduction mechanism of the write operation. However, during the erase operation in Fig. 1a, electrons are injected from the gate to the CSL through the blocking layer. In this case, the barrier height formed by the gate metal and the blocking layer plays an important role in the erase operation. A chemical solution was applied to ensure a clean surface prior to WO_x deposition. A CSL(WO_x) layer was deposited immediately after sample cleaning to minimize the effect of native oxide.

To experimentally calculate the barrier height for each operation, Arrhenius plots were obtained by utilizing the stack as in Fig. 2a and Supplementary Fig. 3b. A linear relationship between $\ln(I/T^2)$ and q/kT was observed through the Arrhenius plot, and it is confirmed that the barrier heights are different between the write and erase operations as shown in Fig. 2b and Supplementary Fig. 3c, respectively. Consequently, because the barrier height is structurally

different for each write and erase operation, there is a difference in the absolute values of write and erase voltages.

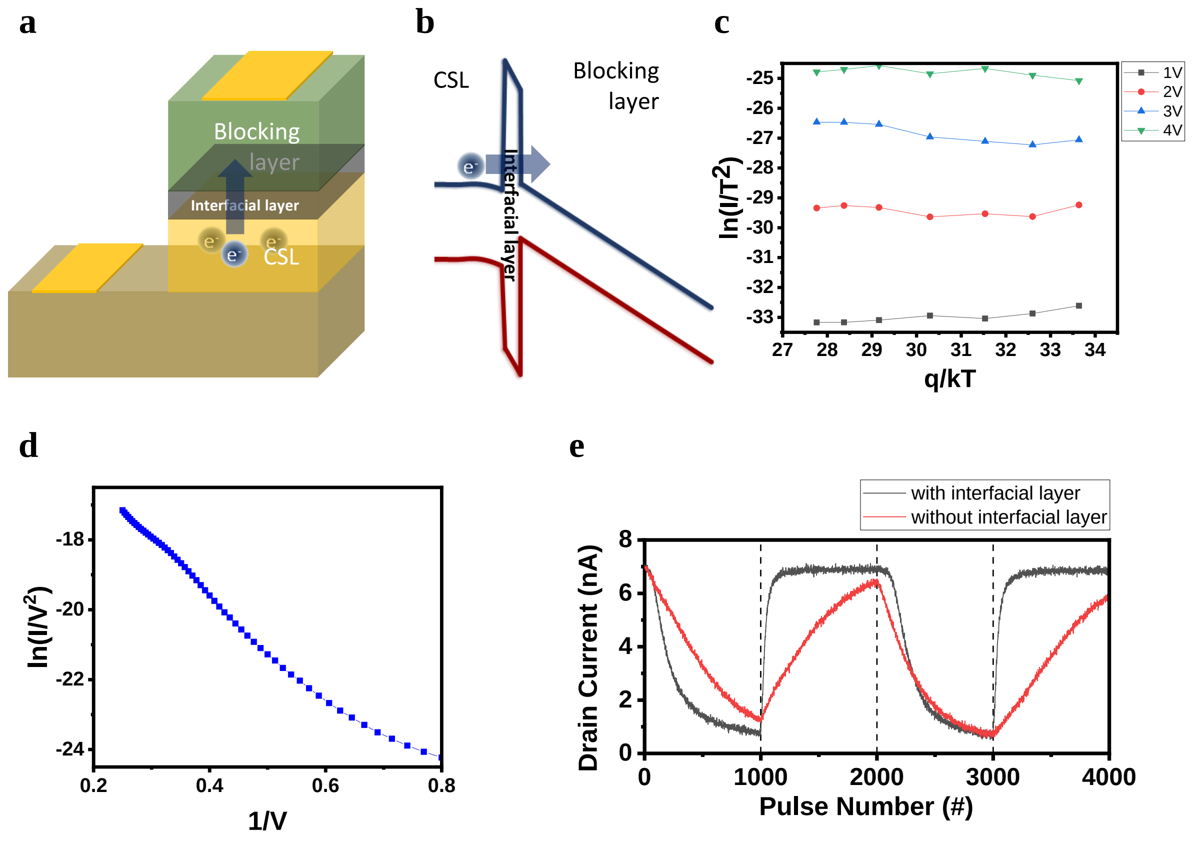

Supplementary Fig. 4 Conduction mechanism for the gate stack with interfacial layer. a Schematic of gate stack with interfacial layer between CSL and blocking layer. **b** Band diagram of changed conduction mechanism by interfacial layer insertion during the write operation (electrons migrated from CSL to blocking layer). **c** The Arrhenius plot for relationship between $\ln(I/T^2)$ and q/kT through the blocking layer with interfacial layer under varied temperature from 345 K to 418 K. **d** Linear relationship between $\ln(I/V^2)$ and $1/V$, where I is the current through the blocking layer with interfacial layer at room temperature. **e** LTP-LTD characteristics of GIFET with and without interfacial layer. The device with interfacial layer shows high nonlinearity (write: 2.5 V, 500 μs, erase: -3 V, 500 μs, read: 1 V, 500 μs).

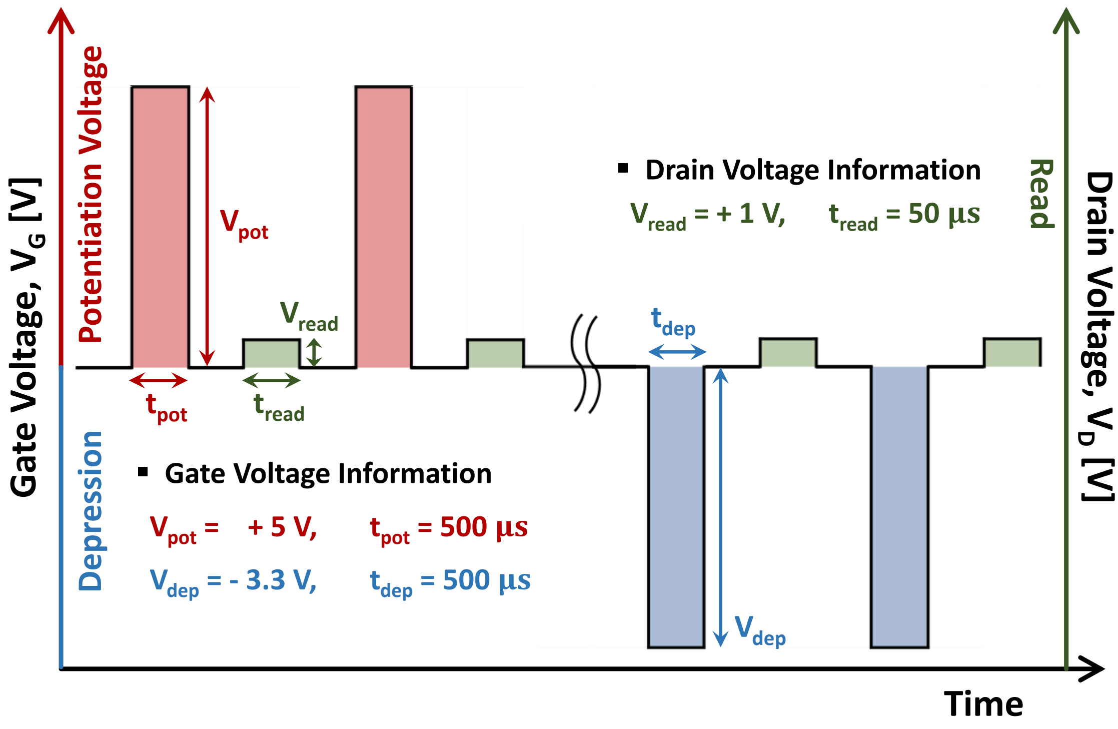

Supplementary Fig. 5 Pulse information for LTP-LTD characteristic. The pulse scheme information for the LTP-LTD characteristic in Fig. 2c. The pulse scheme consists of 1,000 potentiation pulses and 1,000 depression pulses, which are followed by a read pulse after 50 µs each.

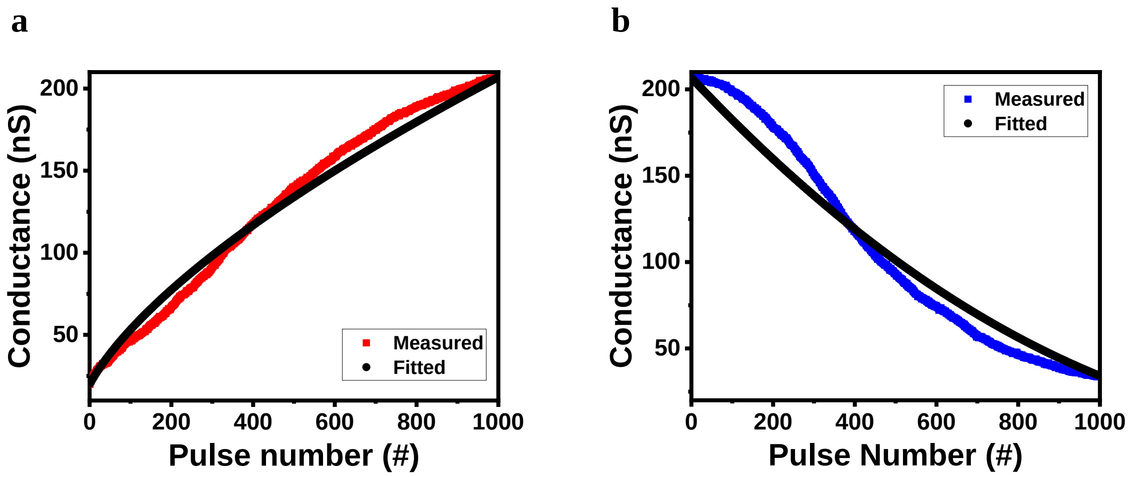

Supplementary Fig. 6 Nonlinearity and asymmetric ratio calculation. **a** The LTP curve of Fig. 2c and fitted curve following the equation of Supplementary note 1. α_{LTP} = 1.53 is calculated, where ideal linearity is represented by 1. **b** The LTD curve of Fig. 2c and fitted curve following the equation of Supplementary note 1. α_{LTD} = 0.47 is calculated.

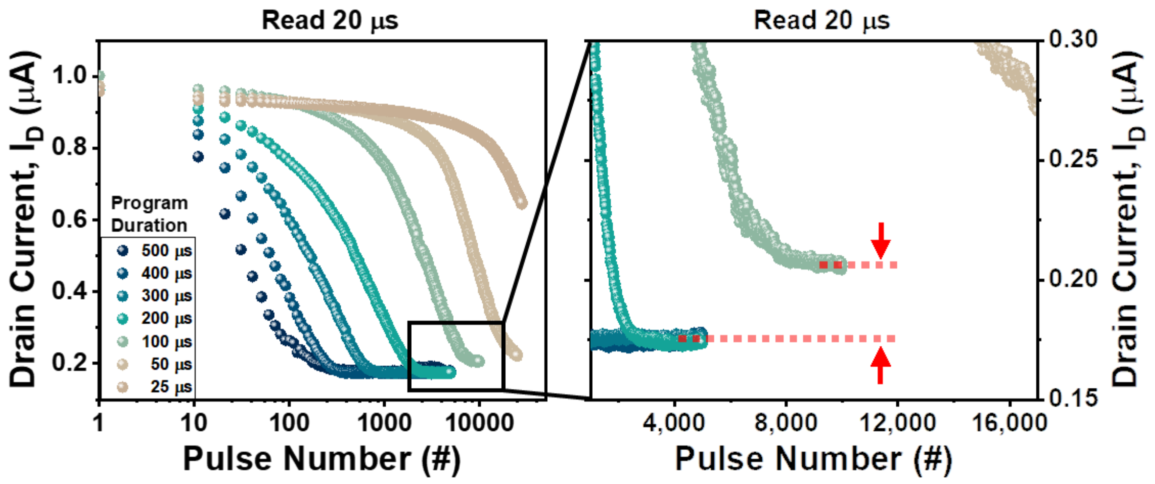

Supplementary Fig. 7 Exploration of the shortest operation time of GIFET. Program operation characteristic under various program pulse duration. The shortest program operation time is investigated as 200 μs under fixed read time. If the program operation time is shorter than 200 μs, regardless of the number of pulses, GIFET does not reach the minimum current level and sacrifices its on/off ratio.

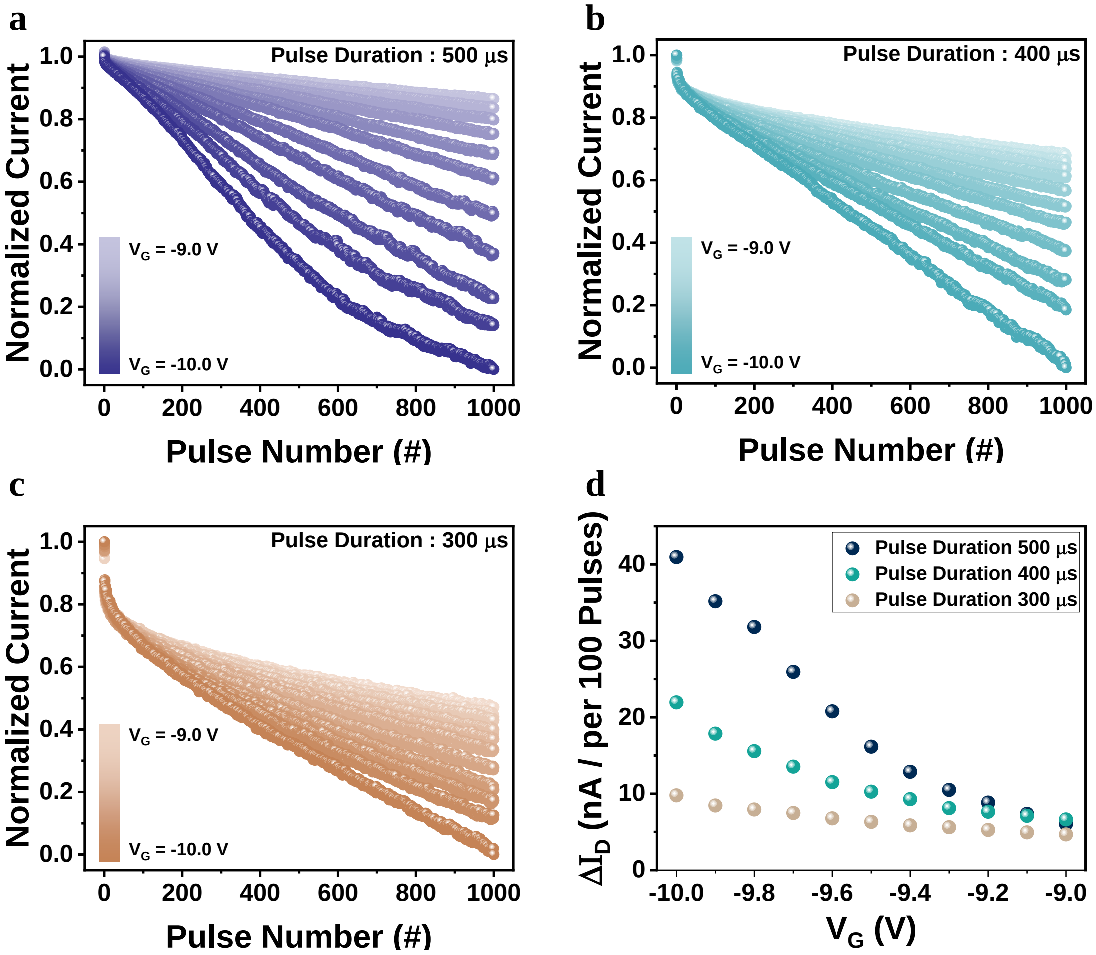

Supplementary Fig. 8 Relationship between pulse amplitude and current change. The current at different depression pulse amplitude from -9 to -10 V at each pulse duration **a** 500 μs, **b** 400 μs, **c** 300 μs. **d** Current change per 100 pulses extracted from Supplementary Fig. 8a-c.

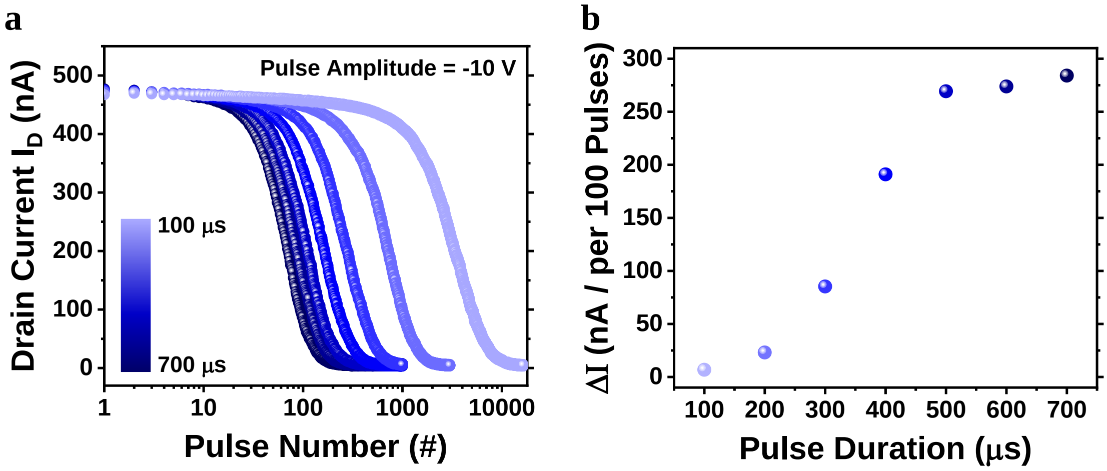

Supplementary Fig. 9 Relationship between pulse duration and current change. a Drain current at different depression pulse duration from 100 to 700 μs at fixed pulse amplitude. **b** Current change per 100 pulses extracted from Supplementary Fig. 9a.

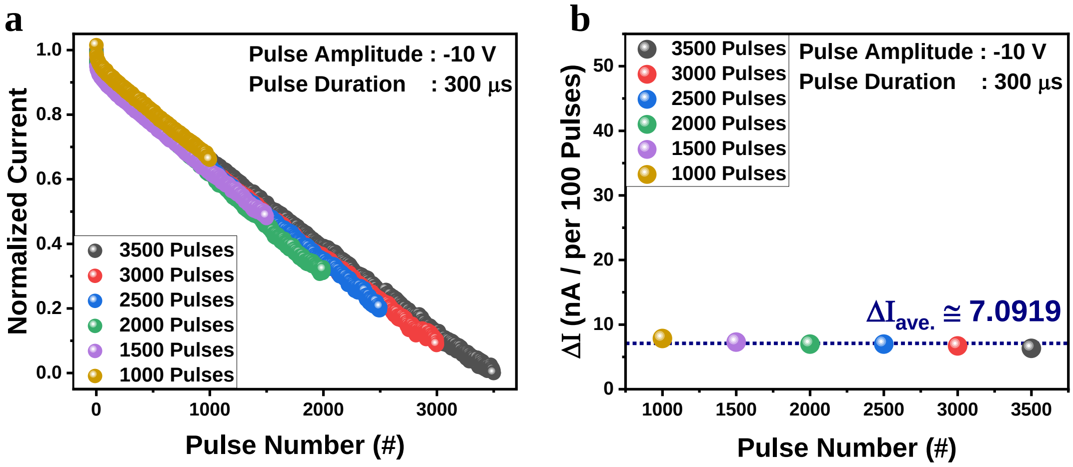

Supplementary Fig. 10 Relationship between pulse number and current change. a Drain current at different depression pulse number from 1,000 to 3,500 at fixed pulse amplitude and duration. **b** Current change per 100 pulses. Linear update values ($\Delta I_{ave.} \cong 7$ nA) are observed even at different pulse number. These values are extracted from Supplementary Fig. 10a.

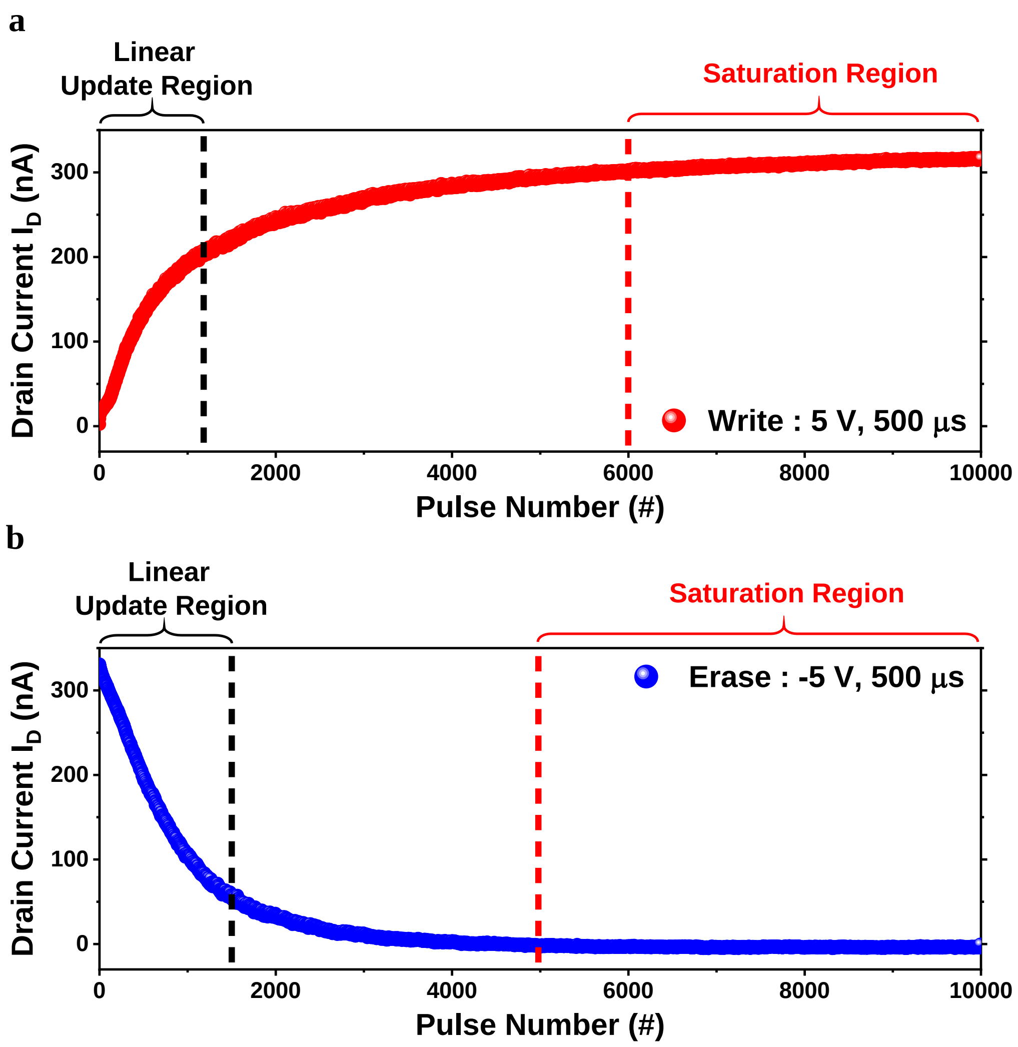

Supplementary Fig. 11 Extended plot of I_D versus pulse number. a Extended I_D versus pulse number plot for write operation (5 V, 500 μs). Drain current linearly increases until approximately 1,200th pulse and starts to saturate from 6,000th pulse. **b** Extended I_D versus pulse number plot for erase operation (-5 V, 500 μs). Drain current linearly decreases until approximately 1,500th pulse and starts to saturate from 5,000th pulse.

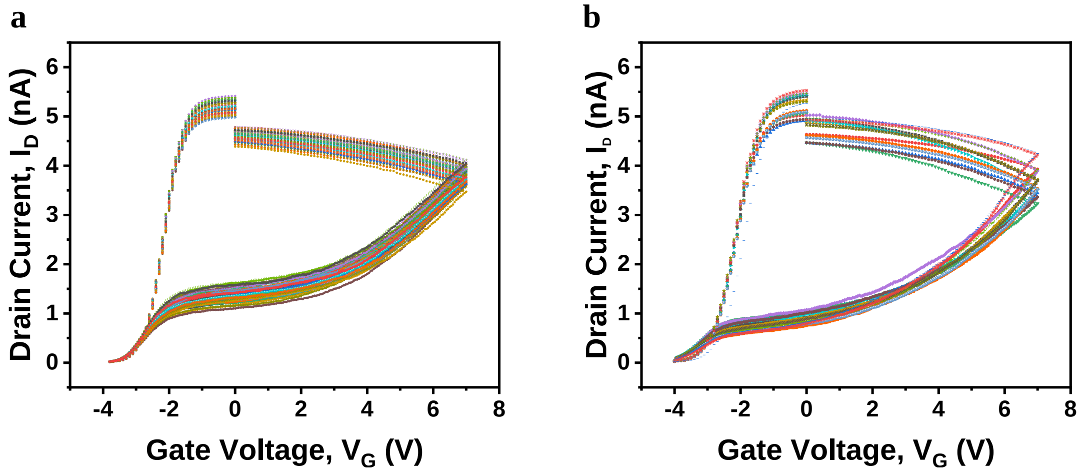

Supplementary Fig. 12 Repeated I-V characteristics of GIFET for cycle-to-cycle and device-to-device measurement. a Repeated cycle-to-cycle I_D-V_G characteristics on a single device. **b** Repeated device-to-device I_D-V_G characteristics for 15 different devices.

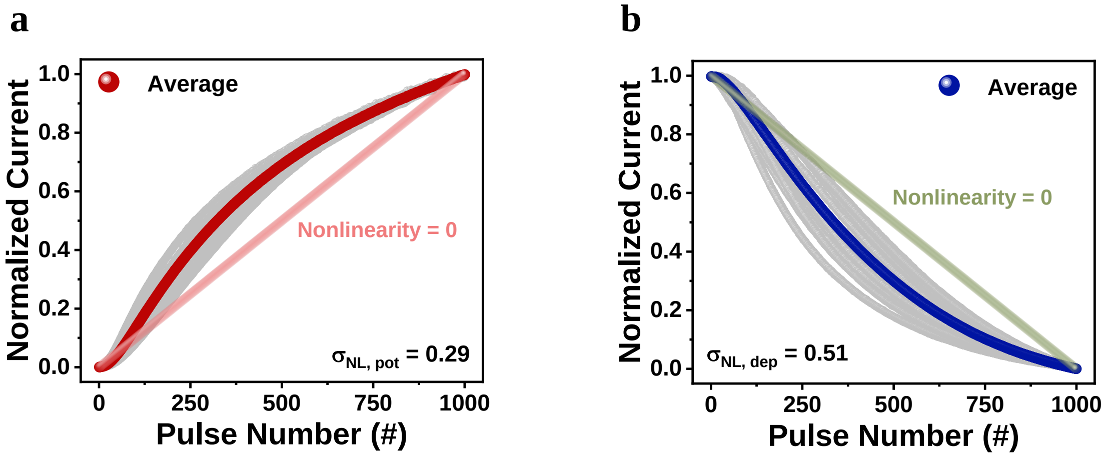

Supplementary Fig. 13. Device-to-device variation of LTP-LTD characteristics. a, b Normalized LTP-LTD characteristics of 15 different devices extracted from Fig. 3d. Nonlinearity was fitted using method from Supplementary Note 2. The average standard deviations of the nonlinearity ($\sigma_{NL,\ pot}$, $\sigma_{NL,\ dep}$) were calculated for simulation of MNIST classification based on Neurosim+.

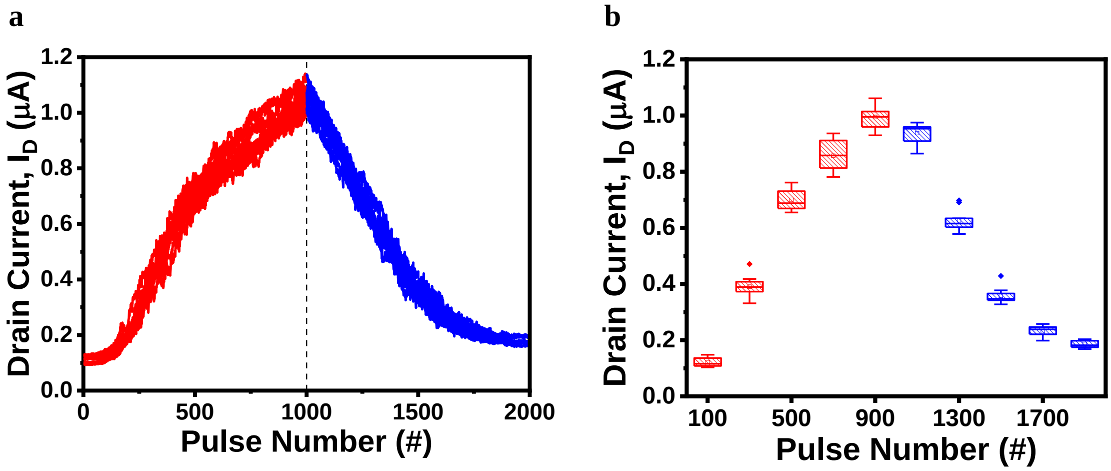

Supplementary Fig. 14 LTP-LTD characteristic of GIFET with highly doped substrate. a Ten repeated LTP-LTD characteristics with microampere level channel current. The pulse scheme of 1,000 write pulses (10 V, 2.5 ms) and 1,000 erase pulses (-3 V, 2.5 ms) was utilized and read pulse (1 V, 2.5 ms) was applied 2 ms after each update pulse. **b** Box plot and variation of the LTP-LTD data in Supplementary Fig. 14a.

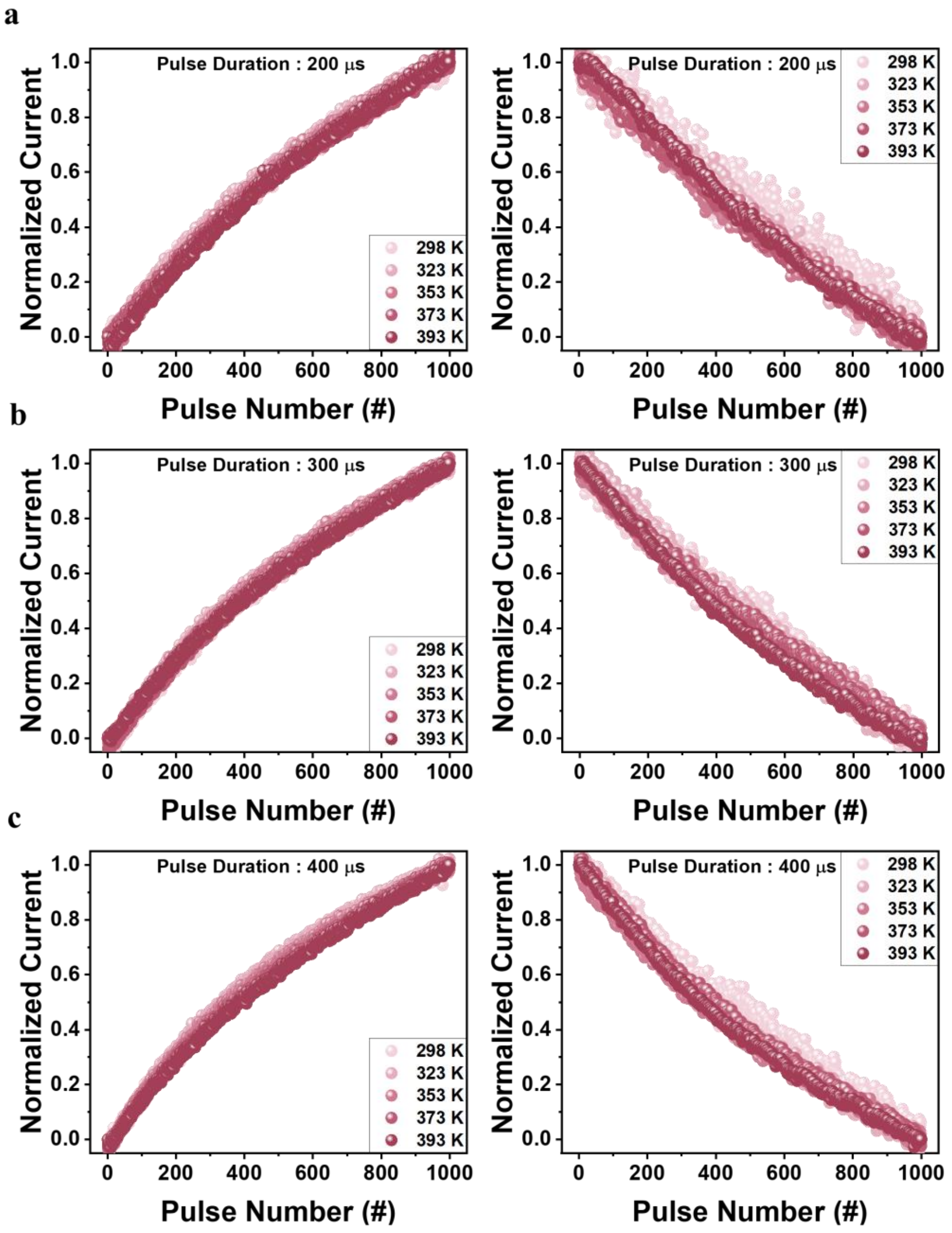

Supplementary Fig. 15 Linearity characteristics at different temperatures. Linearity characteristics on the single device were observed with 1,000 potentiation (4 V) and 1,000 depression (-4 V) gate pulse trains under varied temperature from 293 K to 393 K at each pulse duration **a** 200 μs, **b** 300 μs, and **c** 400 μs.

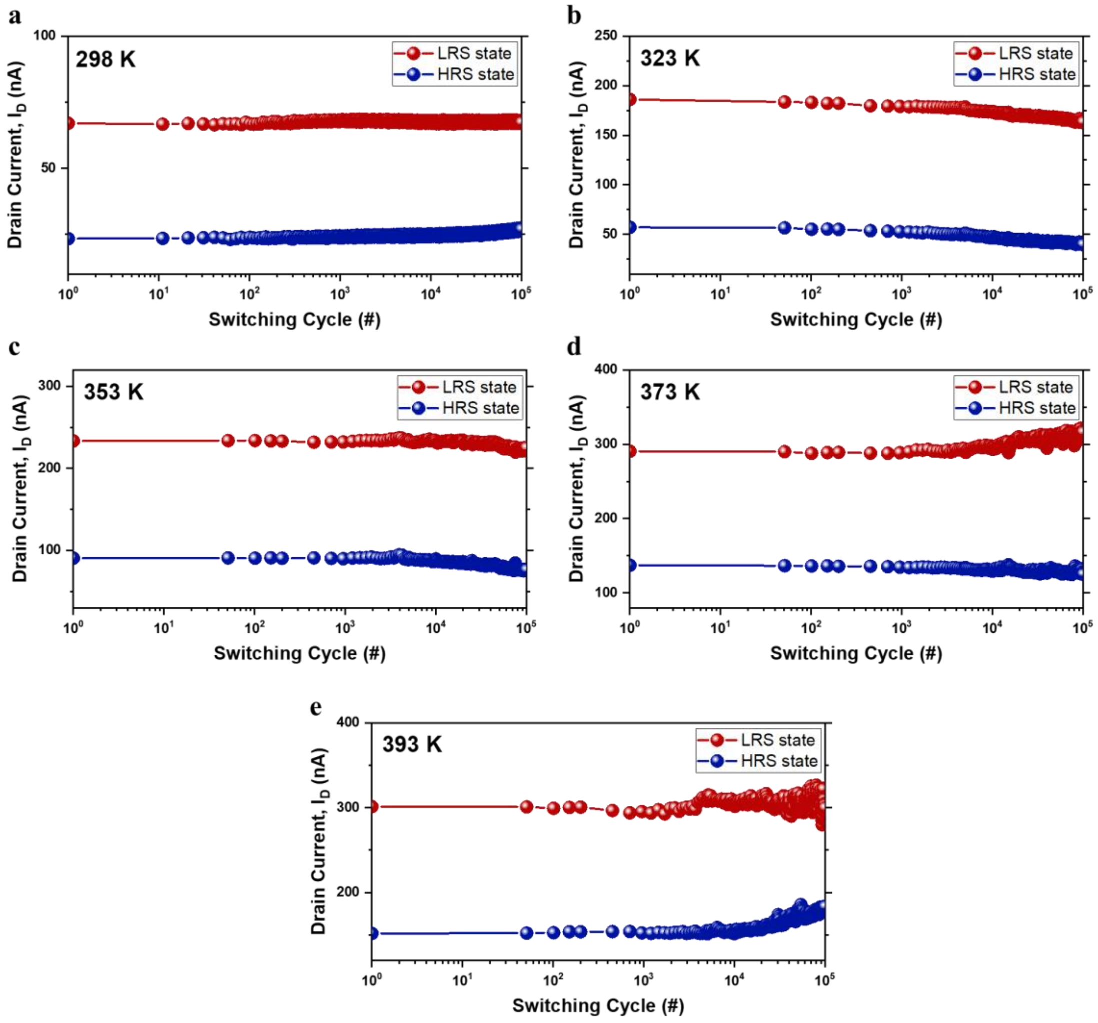

Supplementary Fig. 16 Endurance characteristics in different temperatures. Over 10^5 switching cycles (2×10^6 pulses) were measured. Each switching cycle is composed of 10 potentiation pulses with 6 V, 500 μs and 10 depression pulses with -6 V, 500 μs at **a** 298 K, **b** 323 K, **c** 353 K, **d** 373 K, and **e** 393 K.

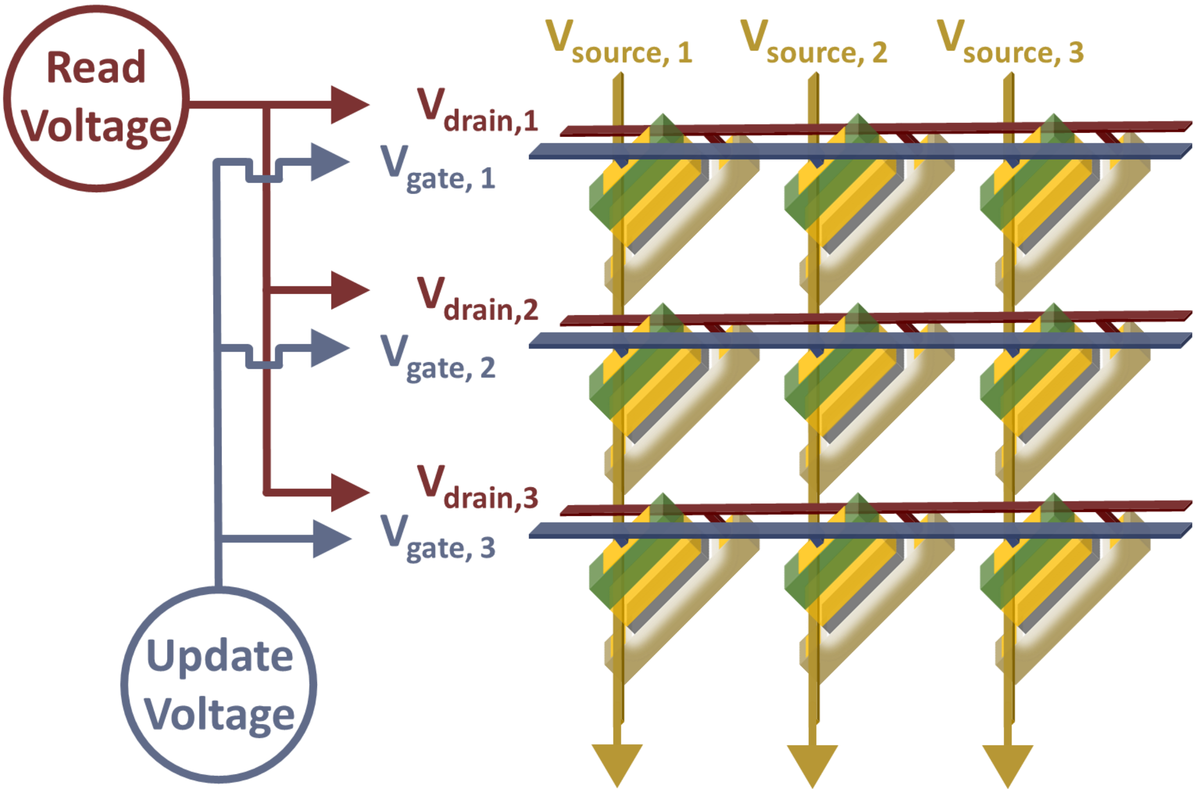

Supplementary Fig. 17 Schematic of GIFET array. The read pulses are applied to the drain of each cell, while the update pulses are applied to the gate of each cell. The output currents are accumulated at the end of the source line.

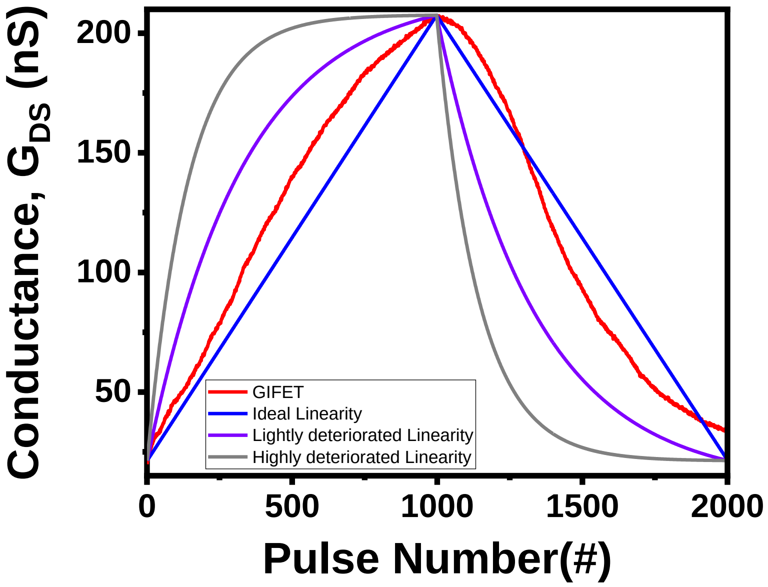

Supplementary Fig. 18 LTP-LTD data with varied linearity based on GIFET parameters for simulation.

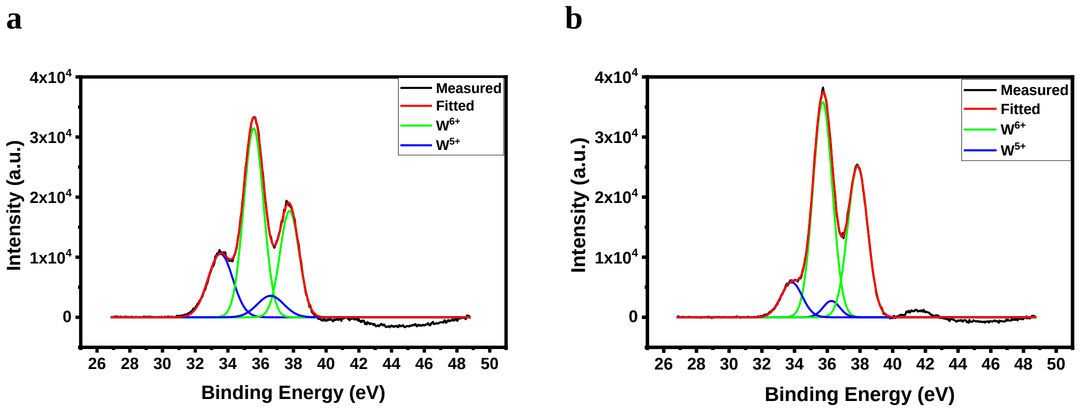

Supplementary Fig. 19 The X-ray Photoelectron Spectroscopy data of the WO_x film. a The XPS data of the WO_x film before annealing. **b** The XPS data of the WO_x film after RTP annealing with 573 K, 10 min.

Supplementary Table 1. Comparison of the accuracy and parameters reflected in the MNIST simulation.

	[8]	[9]	[10]	[11]	[12]	**This work**
Multi Layer Perceptron	400 input<br>100 hidden<br>10 output	400 input<br>100 hidden<br>10 output	400 input<br>100 hidden<br>10 output	400 input<br>100 hidden<br>10 output	400 input<br>100 hidden<br>10 output	**400 input<br>100 hidden<br>10 output**
Conductance states (Potentiation / Depression)	64 / 64	50 / 50	100 / 100	320 / 256	35 / 35	**1,000 / 1,000**
Nonlinearity (Ideal = 0) (LTP / LTD)	-1.54/ -1.76 (Not use identical pulses)	0.07 / -2.42	N/A (Not use identical pulses)	1.22 / -1.75	0.06 / -0.89 (Not use identical pulses)	**0.96 / -0.89**
G_{max}/G_{min}	> 10	> 100	5	> 100	> 10	**> 10**
Temporal Variation	YES	No	YES	YES	No	**YES**
Spatial Variation	YES	No	No	No	No	**YES**
Accuracy	91.1 %	85.88 %	90.6 %	88 %	87 %	**93.17 %**

Supplementary Note 1. The nonlinearity and symmetry calculation.

To calculate the nonlinearity of the GIFET for comparison with other synapse device, the following equation was used with the data extracted from Fig. 2c[4]:

$$G = \begin{cases} ((G_{\mathrm{LRS}}^{\alpha} - G_{\mathrm{HRS}}^{\alpha}) \times w + G_{\mathrm{HRS}}^{\alpha})^{1/\alpha} & if\ \alpha \neq 0 \\ G_{\mathrm{HRS}} \times \left(\frac{G_{\mathrm{LRS}}}{G_{\mathrm{HRS}}}\right)^{w} & if\ \alpha = 0 \end{cases}$$

where G_{LRS} and G_{HRS} are the maximum and minimum conductance in LTP and LTD respectively, α is a parameter reflecting nonlinearity, w is an internal variable 0 to 1. The calculated α with extracted data from Fig. 2c is 1.53 for potentiation and 0.47 for depression. These values are close to 1, which represents ideal linear and symmetric conductance change.

To figure out the asymmetric ratio of the GIFET, the following equation was used with the data extracted from Fig. 2c[5]:

$$AR = \frac{\max|G_{\mathrm{P}}(n) - G_{\mathrm{D}}(n)|}{G_{\mathrm{P}}(PN_{\max}) - G_{\mathrm{D}}(PN_{\max})}\ for\ n = 1\ to\ PN_{\max}$$

where $G_{\mathrm{P}}(\mathrm{n})$ and $G_{\mathrm{D}}(\mathrm{n})$ are the channel conductance at n^{th} potentiation and depression, and $PN_{\max}$ is the number of potentiation or depression pulses. The asymmetric ratio of the GIFET is calculated as 0.29.

Supplementary Note 2. Nonlinearity in the simulation.

The spatial variation was defined as the standard deviation of the nonlinearity baseline in the NeuroSim+ simulation. To reflect the device-to-device of the GIFET in the MNIST classification simulation, the following equation was used to calculate the nonlinearity of the GIFET in the simulation:

$$G_{\mathrm{LTP}} = B\left(1 - e^{(\frac{P}{A})}\right) + G_{\min}$$

$$G_{\mathrm{LTD}} = B\left(1 - e^{(\frac{P - P_{\max}}{A})}\right) + G_{\max}$$

$$B = (G_{\max} - G_{\min})/\left(1 - e^{\frac{-P_{\max}}{A}}\right)$$

where G_{LTP} and G_{LTD} are the conductance of LTP and LTD, respectively. G_{max}, G_{min}, and P_{max} were extracted directly from Supplementary Fig. 13a, b. A is a parameter that controls the nonlinear behavior of the weight update. This value being close to zero, which represents the ideal linear conductance change[6].

Supplementary Note 3. The GIFET with high doping channel layer.

The device of Supplementary Fig. 14 was based on 145 nm n-type high doping channel layer, which created by ion implantation with dose 5×10^{14} cm^{-2}, energy 60 keV, phosphorus and annealing with 1,273 K, N_2 atmosphere, 30 s. 25 nm gate oxide was deposited on top of the Si layer and 150 nm WO_x were utilized as the CSL. The blocking layer a-Si:H 50nm and Ti/Au gate metal was used.

Supplementary Note 4. The array operation of the GIFET

Supplementary Fig. 17 presents the schematic of GIFET based crossbar array for the neural network. As presented, the vector-matrix multiplication (VMM) is able to be conducted through the crossbar array. Input vector, represented by the number of successive read pulses, apply on each bit line. These input pulses are multiplied by conductance stored at each cell connected to the bit line following ohm's law, and the output currents are accumulated on the connected source line following Kirchhoff's current law, finishing the VMM. The weight of each cell can be updated by applying write or erase voltage to the gate.

Supplementary Note 5. The XPS data of WO_x film

To inspect the influence of Rapid Thermal Processing on the stoichiometry of the WO_x layer, XPS measurements were conducted. The 90 nm WO_x films were deposited on cleaned SiO_2 (100 nm)/Si substrate. One of the two 2 cm×2 cm samples was annealed with RTP at 573 K for 10 mins in O_2 atmosphere. Supplementary Fig. 19 shows the XPS data of the sample without annealing (Supplementary Fig. 19a) and with annealing (Supplementary Fig. 19b). The data was calibrated with carbon C_{1s} XPS peak to be located at 284.8 eV[7]. As presented in Supplementary Fig. 19, the W^{5+} intensity of the WO_x film was reduced after annealing, which suggests an increase of W^{6+}. The computed stoichiometry from surface atomic ratio increases from $WO_{2.60}$ to $WO_{2.75}$ after annealing, which implies increasing barrier height.

References for Supplementary Information

1. Robertson, J. High dielectric constant oxides. *EPJ Appl. Phys.* **28**, 265–291 (2004).

2. Deb, S. K. Optical and photoelectric properties and colour centres in thin films of tungsten oxide. *Philos. Mag.* **27**, 801–822 (1973).

3. Kalema, V. N., Aljishi, S., Dawson, R. M. A., Slobodin, D. & Wagner, S. The dielectric constants of a-Si, Ge: H, F alloys. *Mater. Lett.* **4**, 320–322 (1986).

4. Jang, J.-W., Park, S., Jeong, Y.-H. & Hwang, H. ReRAM-based synaptic device for neuromorphic computing. In *2014 IEEE International Symposium on Circuits and Systems (ISCAS)* 1054–1057 (IEEE, 2014).

5. Yu, J. M. et al. All-Solid-State Ion Synaptic Transistor for Wafer-Scale Integration with Electrolyte of a Nanoscale Thickness. *Adv. Funct. Mater.* **2010971**, 1–10 (2021).

6. Chen, P.-Y., Peng, X. & Yu, S. NeuroSim+: An integrated device-to-algorithm framework for benchmarking synaptic devices and array architectures. *IEEE Int. Electron Devices Meeting (IEDM)* (IEEE, San Francisco, USA, 2017).

7. Korkos, S. et al. XPS analysis and electrical conduction mechanisms of atomic layer deposition grown Ta_2O_5 thin films onto p-Si substrates . *J. Vac. Sci. Technol. A* **38**, 032402 (2020).

8. Kim, M. K. & Lee, J. S. Ferroelectric Analog Synaptic Transistors. *Nano Lett.* **19**, 2044–2050 (2019).

9. Yu, R. et al. Electret-based organic synaptic transistor for neuromorphic computing. *ACS Appl. Mater. Interfaces* **12**, 15446–15455 (2020).

10. Wang, L. et al. Exploring ferroelectric switching in α-In_2Se_3 for neuromorphic computing. *Adv. Funct. Mater.* **30**, 2004609 (2020).

11. Chung, W., Si, M. & Peide, D. Y. First demonstration of Ge ferroelectric nanowire FET as synaptic device for online learning in neural network with high number of conductance state and G_{max}/G_{min}. In *2018 IEEE International Electron Devices Meeting (IEDM)* 12–15 (IEEE, 2018).

12. Chou, Y.-C. et al. Neuro-inspired-in-memory computing using charge-trapping

memtransistor on germanium as synaptic device. *IEEE Trans. Electron Devices* **67**, 3605–3609 (2020).
